# Cervical Adenocarcinoma In Situ in Young Nulliparous Patient with Persistent ASC-US and Multiple-Type HPV Infections Without HPV 16 and 18 Types—Case Report

**DOI:** 10.3390/diagnostics16040617

**Published:** 2026-02-20

**Authors:** Nikola Milic, Marija Varnicic Lojanica, Stefan Ivanovic, Milica Ivanovic, Katarina Ivanovic, Nikola Jovic

**Affiliations:** 1 Užice General Hospital, 31000 Užice, Serbia; 2 Obstetrics and Gynecology Clinic “Narodni Front”, 11000 Belgrade, Serbia; 3 Clinic for Gynecology and Obstetrics, University Clinical Center of Serbia, 11000 Belgrade, Serbia; 4 Department of Gynecology and Obstetrics, Faculty of Medical Sciences, University of Kragujevac, 34000 Kragujevac, Serbia; 5 University Clinical Center Kragujevac, Zmaj Jovina 30, 34000 Kragujevac, Serbia

**Keywords:** adenocarcinoma in situ, cervix uteri, human papillomavirus, ASC-US

## Abstract

The most severe premalignant lesion of glandular epithelium of the cervix is adenocarcinoma in situ (AIS). In most cases it is associated with persistent human papillomavirus (HPV) infection and most often occurs in women in the fourth decade of life. In most high-income countries, primary screening has shifted to HPV testing, while cytology is used for patient triage. Even with current robust screening protocols, their sensitivity for glandular lesions remains limited. Diagnosis of AIS obtained by biopsy, brushing or curettage is confirmed by excisional methods and pathohistological verification. Therapy depends on the patient’s lifestyle and reproductive age. In our case, we present a nulliparous patient with persistent ASC-US, multiple-type HPV infection without HPV 16 and 18 types, and AIS which was diagnosed after conization, follow-up and two biopsies with curettage of cervical canal. Our case report highlights limitations in detection of glandular lesions and need for caution in patients with persistent and seemingly low-grade cytological abnormalities, notably in young patients with high-risk HPV types.

## 1. Introduction

Adenocarcinoma in situ (AIS) of the uterine cervix is defined as the most severe premalignant lesion of the glandular epithelium, characterized by the absence of basement membrane invasion, and represents a direct precursor to invasive cervical carcinoma, a potentially life-threatening disease [1]. It is a very rare entity in the general population of women, and most cases are diagnosed in fourth decade. However, in recent years there has been an increased incidence rate in younger patients, notably patients in their twenties and thirties. Adenocarcinomas account for approximately 15–25% of all cervical cancers [2,3]. In most cases, AIS is associated with persistent HPV infection, and according to the available literature, HPV represents the main etiological factor in cervical carcinogenesis [4,5]. In invasive cervical carcinoma the most common HPV types include types 16, 18, 45, 31, 33, 58, 52, 35, 59, 56, 51, 68, 39, 82, 73, 66 and 70. In squamous carcinoma the most dominant HPV type is HPV-16 (46–63%), followed by HPV-18 (10–14%), HPV-45 (2–8%), HPV-31 (2–7%) and HPV-33 (3–5%). The prevalence of HPV infection is significantly lower in adenocarcinoma (76.4%) than in squamous carcinoma (87.3%), and the most dominant HPV type is HPV-18 (37–41%), followed by HPV-16 (26–36%) and HPV-45 (5–7%) (Table 1) [6]. In the scientific community there is still a dispute whether HPV permanently remains in basal cells or is eliminated over time. Authors acknowledge that persistent HPV infection includes two sequentialpositive tests within 6–12 months [7]. In everyday Pap test sampling, ASC-US is a common finding, and it is important to recognize because AIS can be present with similarly minimal and non-specific abnormalities [8]. Despite continuous improvements in cervical cancer screening strategies, the detection of glandular lesions remains more challenging compared to squamous precursors. Recent advances in molecular and translational research have further highlighted the biological heterogeneity and complexity of HPV-associated cervical carcinogenesis. Contemporary studies emphasize the diversity of oncogenic pathways, viral–host interactions, and tumor microenvironment characteristics involved in the development and progression of cervical and endocervical neoplasia. Their findings indicate that cervical carcinogenesis cannot be fully explained solely by the presence of dominant HPV genotypes or cytological abnormalities. Despite significant progress in molecular understanding, translation of these insights into routine clinical screening remains limited, particularly in the early detection of glandular lesions. Consequently, clinically significant pathology may remain undetected even in patients undergoing regular cytological and HPV-based surveillance. This growing body of evidence further underscores the need for heightened clinical vigilance and more refined diagnostic strategies in patients with persistent abnormalities and non-16/18 high-risk HPV infections [9,10,11,12]. Pathohistological verification provides definitive diagnosis, while decision about whether to perform biopsy or curettage is not easily made, especially in younger, nulliparous patients, suchas in our case [13]. This difficulty arises because such invasive diagnostic steps frequently fall outside the routine screening and triage algorithms recommended by most contemporary international guidelines, as was the case in our patient [13,14,15].

The aim of this case report is to present a rare case of AIS in a young patient (in her early twenties) and to highlight the limitations of cervical cancer screening tests in the detection of glandular lesions.

## 2. Case Presentation

**Figure 1:**
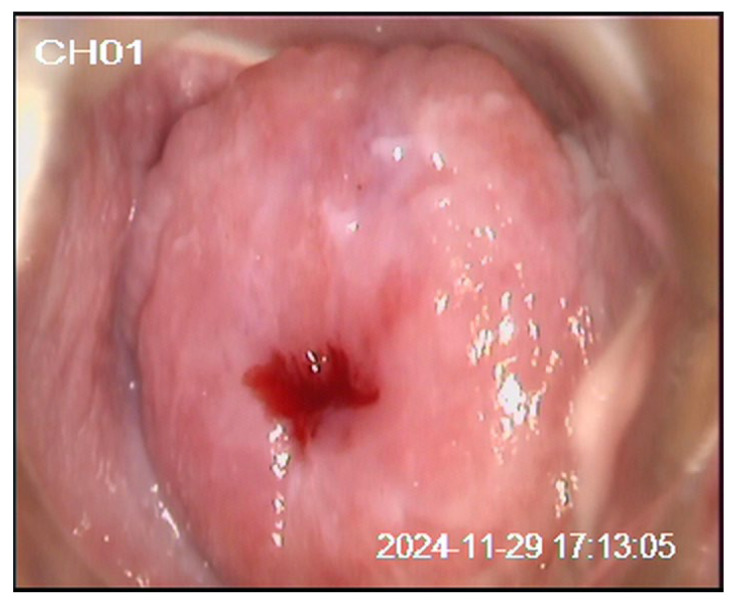
Native appearance of the cervix following conventional colposcopic examination.

**Figure 2:**
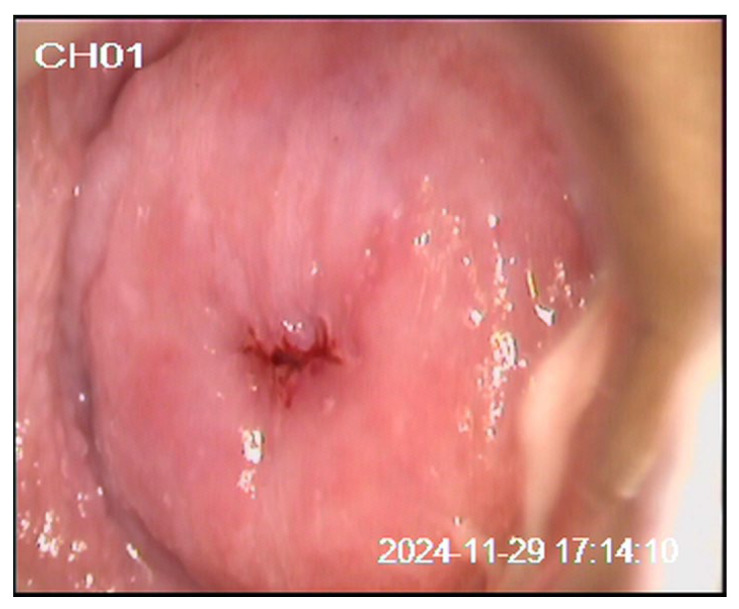
Colposcopic appearance of the cervix after application of 3% acetic acid.

**Figure 3:**
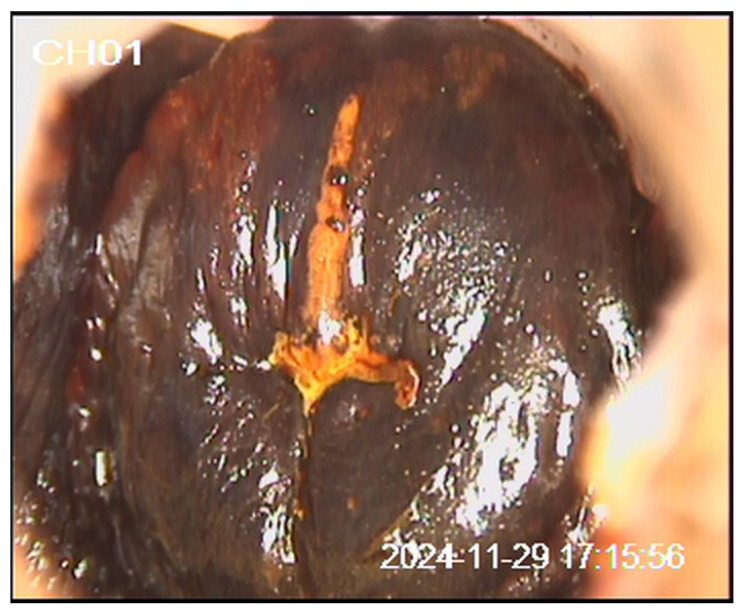
Cervical appearance after application of Lugol’s iodine solution.

**Figure 4:**
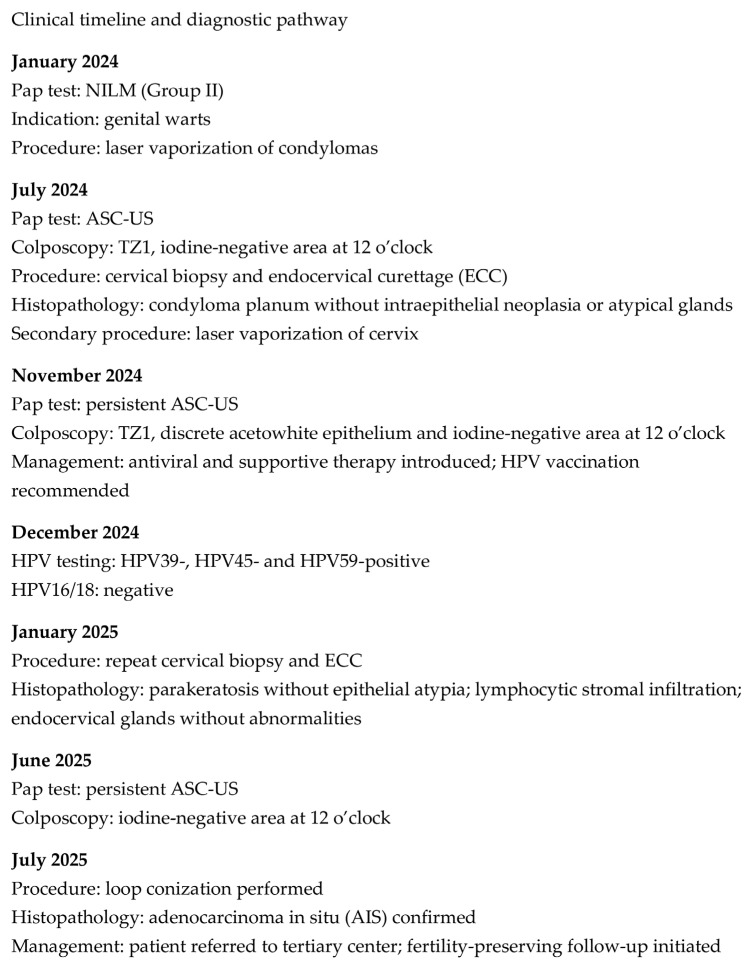
Diagnostic pathway and clinical timeline of the patient. The figure illustrates the sequential cytological, colposcopic, histopathological, and therapeutic steps taken from January 2024 to July 2025.

We present a 20-year-old patient with no prior history of delivery or miscarriage. Her menstrual cycles were regular, with menarche at the age of 12. She denied allergies to food or medications, previous surgeries, or significant medical illnesses, and her family history was unremarkable. The patient was a smoker (up to 20 cigarettes per day), had been sexually active for three years, and had not received HPV vaccination. The patient first presented in January 2024 due to the presence of genital warts two years after her first sexual contact. A Papanicolaou (Pap) test was performed (classified as group II), and laser vaporization of the genital warts was carried out. In July 2024, she was re-examined for small condylomas. The Pap test was designated as ASC-US according to the Bethesda classification. Colposcopy revealed a type 1 transformation zone (TZ1) and an iodine-negative area at the 12 o’clock position. A cervical biopsy and endocervical curettage (ECC) confirmed condyloma planum without intraepithelial neoplasia or atypical glands, and laser vaporization of the cervix was performed. In November 2024, follow-up Pap testing and colposcopy demonstrated TZ1 with discrete acetowhite epithelium and an iodine-negative area at the 12 o’clock position, while the Pap test again showed ASC-US (Figure 1, Figure 2 and Figure 3). Antiviral and supplement therapy were introduced and HPV vaccination was recommended. In December 2024, PCR testing for HPV was performed, confirming negativity for HPV types 16 and 18. At the same time, pooled testing for high-risk HPV types was positive for genotypes included in the extended high-risk panel (HPV 31, 33, 35, 39, 45, 51, 52, 56, 58, 59, 66, and 68). Subsequent targeted genotyping enabled precise identification of the present types, confirming HPV 39, HPV 45, and HPV 59. In January 2025, follow-up biopsy and ECC were performed, revealing parakeratosis without cervical epithelial atypia, lymphocytic stromal infiltration, and glandular cells of the endocervical canal without abnormalities. In June 2025, repeat Pap testing and colposcopy showed persistent ASC-US and an iodine-negative area at the 12 o’clock position. The previously described targeted biopsies were obtained from the colposcopically suspicious area at the 12 o’clock position, with routine ECC performed at each evaluation. The biopsy specimens measured approximately 3 mm in depth and diameter. We believe that the previously AIS-negative histopathological findings may be explained by the specific anatomy and glandular origin of AIS, which frequently develops along endocervical crypts and may exhibit a focal and discontinuous (“skip lesion”) growth pattern beyond the reach of superficial biopsies and standard endocervical sampling. In July 2025, loop electrosurgical conization was subsequently performed. Histopathological and immunohistochemical analysis confirmed focal AIS. Strong p16INK4a positivity was observed within the dysplastic endocervical glands, while Ki-67 showed diffuse proliferative activity, supporting HPV-associated glandular neoplasia and confirming the diagnosis of AIS. Fertility-preserving close follow-up for a minimum of five years in a tertiary center was recommended (Pap test, colposcopy, and repeat HPV testing as part of long-term surveillance). The diagnostic pathway and overall clinical timeline of the patient are summarized in Figure 4.

## 3. Discussion

### 3.1. Pathophysiology of Cervical Adenocarcinoma In Situ

AIS represents a terminal and the most severe precancerous lesion, with high malignant potential, on the pathway of glandular carcinogenesis of the uterine cervix [1,3]. Its key feature is the replacement of the normal endocervical glandular epithelium with atypical, mitotically active cells, with preserved integrity of the basement membrane [1,10]. Unlike squamous intraepithelial lesions, which arise in the transformation zone and are easily detected by colposcopic examination and cytological analysis, AIS originates from the deeply located glandular epithelium of the endocervix, encompassing endocervical crypts and glandular structures, because of which the lesion often develops outside the scope of direct visualization [1,3,16]. The molecular pathogenesis of AIS is predominantly mediated by persistent infection with high-risk HPV [5,6]. Integration of the viral genome into the DNA of the host epithelium represents a key oncogenic event, which leads to persistent expression of the E6 and E7 oncoproteins [5]. These viral proteins promote degradation of the tumor suppressor proteins p53 and retinoblastoma (Rb), which results in genomic instability and deregulated control of the cell cycle [5,6]. These molecular changes initiate dysplastic transformation of the glandular epithelium and clonal expansion along the endocervical glandular ducts [1,3,17]. Growth patterns of glandular lesions are often multifocal and discontinuous, leading to so-called “skip lesions”, which further hinder detection and complete excision [18,19,20]. Overexpression of immunohistochemical surrogate biomarkers, such as p16^INK4a^, reflects HPV-associated oncogenesis and has a key role in establishing the diagnosis, particularly in cases with concordant morphological findings [7,15]. A deep and insidious pattern of spread, in combination with discontinuous growth and absence of involvement of the surface epithelium, explains the frequent failure of conventional screening methods in detecting AIS [1,8,20]. Accordingly, histopathological evaluation of excisional specimens remains the diagnostic gold standard [8,17].

### 3.2. Limitations of Current Cervical Cancer Screening Strategies for Glandular Lesions

Despite significant progress in the prevention of cervical cancer, existing screening strategies are still primarily adapted to squamous intraepithelial lesions [16,21,22]. Cytology, including both conventional and LBC, shows limited sensitivity for glandular lesions, because the cytological features of AIS are often minimal, nonspecific, or completely absent [8,23,24]. Findings reported as ASC-US, although common and usually considered low-risk, may conceal underlying glandular lesions, leading to a false sense of security and delayed diagnosis [8,25]. Primary HPV testing has significantly improved the detection of high-grade squamous intraepithelial lesions; however, its sensitivity for glandular pathology remains relatively limited [16,24,26]. Although HPV infection and AIS are closely associated with cervical adenocarcinoma, a subset of cases is attributed to high-risk HPV genotypes other than HPV16 and 18, most commonly those belonging to the alpha-7 species, which include HPV18, HPV39, HPV45, HPV59, HPV68, HPV70, HPV85 and HPV97 [5,17,18]. Multiple HPV infections without HPV-16/-18 positivity represent a rare but documented etiopathological factor [17,18]. Consequently, screening strategies that are predominantly focused on HPV 16/18 positivity may insufficiently capture the risk associated with non-16 high-risk infections [18,24,27]. Colposcopy, even when combined with targeted lesion mapping and guided biopsy, remains an inherently subjective method with limited ability to assess the endocervical canal [28,29,30]. As a result, glandular lesions such as AIS may escape detection despite repeated colposcopic examinations and biopsies [8,20]. This diagnostic limitation reveals a significant shortcoming in existing screening algorithms and emphasizes the importance of increased clinical vigilance in patients with persistent abnormal findings and high-risk HPV infection, even in the absence of guideline-defined indications for invasive diagnostic intervention [16,24,27]. AIS represents a particular diagnostic challenge, as it is difficult to detect even with cytology and HPV testing [8,24]. Although common in everyday clinical practice, ASC-US conceals a small number of glandular lesions [17,25]. Unlike squamous lesions, AIS typically originates from endocervical crypts and is located deep within the endocervical canal, which significantly limits visualization during colposcopic examination [1,20]. The anatomy of the uterine cervix often prevents adequate visualization of the entire transformation zone and the endocervical epithelium, thereby reducing colposcopic sensitivity for glandular lesions [1,30,31]. An additional diagnostic challenge is represented by “skip lesions”, which reflect the multifocal and discontinuous nature of glandular disease [19,20,32]. Approximately 15% of cases show discontinuous lesions at other sites within the endocervical epithelium, even when AIS is focally identified at a single site [20,22,33]. These characteristics increase the risk of false-negative colposcopic biopsies and represent a key limitation of current screening and diagnostic algorithms for the early detection of AIS [8,21,34]. The Pap test, introduced into routine clinical practice during the second half of the 20th century, remains one of the most effective screening tools for the early detection of cervical cancer [13,22]. Cytology is widely used in both conventional and liquid-based forms [22]. HPV testing was introduced later as a primary screening method, mainly in high-income countries due to financial feasibility [23,24]. However, its limited effectiveness in the detection of glandular lesions has been documented, which emphasizes the need for additional strategies [8,24]. Bansal and colleagues showed moderate specificity of conventional (60%) and liquid-based cytology (64%) in the detection of glandular abnormalities, with no significant difference between these two methods [25]. Both methods show moderate specificity and low sensitivity, which emphasizes the importance of HPV co-testing [8,25]. According to contemporary international guidelines, primary HPV testing represents the backbone of cervical cancer screening, with cytology serving as a triage tool in HPV-positive patients [16,24,31,32,33]. Colposcopy is considered an additional diagnostic method reserved for selected high-risk cases [26,27,30]. The 2019 ASCCP consensus guidelines use a risk-based approach, applying HPV genotyping, cytology, and prior histopathology to calculate the immediate risk for CIN3+, with colposcopy indicated when the risk exceeds 4% [26,27,28]. The Australian national cervical cancer screening program similarly relies on primary HPV DNA testing, with cytology as a triage tool and colposcopy reserved for high-risk scenarios [29,30]. A review of the recommendations of major professional organizations, including the American Cancer Society (ACS), the American Society of Clinical Oncology (ASCO), the World Health Organization (WHO), Cancer Council Australia (CCA), and European guidelines, consistently supports HPV testing as the primary method of cervical cancer screening [22,29,30,31,32,33,34,35]. After biopsy, excisional procedures and histopathological analysis are necessary for the definitive diagnosis of AIS [8,17]. Cold knife conization or electrosurgical conization is recommended as the excision technique. The electrosurgical knife may be used by an experienced gynecologist, with preservation of specimen margins ensured [8,20]. The specimen length should be at least 10 mm, and up to 20 mm in patients who have completed reproduction [8,20]. Treatment depends on age, reproductive desires, and margin status [8,21,27]. Fertility-preserving follow-up protocols that include HPV testing and cytology, with or without colposcopy and endocervical curettage, are increasingly supported by evidence [8,19,21]. HPV positivity during follow-up is recognized as a strong predictor of recurrence [16,36,37]. Giannella and colleagues did not show a statistically significant difference in recurrence rates or long-term survival between conservative treatment with conization and definitive treatment with hysterectomy in adenocarcinoma in situ and microinvasive adenocarcinoma of the uterine cervix, although the conservative approach was associated with a higher rate of local recurrence (7.1%), with preserved long-term survival [37]. Despite the implementation of contemporary screening programs, HPV vaccination coverage remains insufficient, particularly in low-income countries [32,33]. A Japanese cohort study showed a significant decrease in the prevalence of HPV-16 and -18 following high vaccination coverage [38]. The HPV-IMPACT program in the United States reported an 80% reduction in the incidence of CIN3 and AIS among women in their early twenties [39]. A large meta-analysis published in *The Lancet*, which included more than 60 million individuals from 14 high-income countries, confirmed the high effectiveness of HPV vaccination in the prevention of severe cervical lesions [40]. Although HPV types 16 and 18 were not detected in the present case, currently available nonavalent HPV vaccines provide coverage against several additional high-risk genotypes, including HPV 31, 33, 45, 52, and 58. Therefore, vaccination may still provide partial protection and reduce the risk of future HPV-related lesions and reinfection, even in patients with pre-existing HPV infection, as supported by evidence demonstrating substantial population-level impact and herd effects following the introduction of HPV vaccination programs [40].

### 3.3. Epidemiology and Age Distribution of Adenocarcinoma In Situ

AIS and invasive cervical cancer are predominantly diagnosed in women in their third to fifth decades of life, with a mean age at diagnosis of approximately 50–53 years. Epidemiological data indicate that fewer than 1–2% of cervical cancer cases occur in women younger than 25 years, while occurrence in adolescent and early reproductive age remains exceptionally uncommon [2,15,17]. The low incidence of cervical cancer and its precursor lesions during adolescence and early reproductive age constitutes the basis of international guidelines that do not recommend routine screening in this population [13,31,33]. However, emerging epidemiological evidence indicates a shift toward a younger age at diagnosis, due to changes in sexual behavior, the dynamics of HPV exposure, and deficiencies in screening coverage [2,15,17,33]. Because AIS is uncommon in very young women, clinicians have a lower degree of suspicion, which contributes to a delay in establishing the definitive diagnosis [13,15,16]. Available evidence indicates that affected patients often share risk factors such as early onset of sexual activity, persistent HPV infection and lack of HPV immunization [14,16,33,38]. Cigarette smoking is a well-established cofactor in HPV-related cervical carcinogenesis. Tobacco-related carcinogens have been shown to impair local immune response, promote viral persistence, and facilitate progression from HPV infection to high-grade intraepithelial lesions and glandular neoplasia. Recent evidence further indicates that prolonged smoking significantly promotes persistence and progression of high-risk HPV infection and is associated with higher rates of cytological and histopathological abnormalities. In HPV-positive women, abnormal histopathological findings have been reported to be more than twofold higher among smokers, particularly with increasing cumulative tobacco exposure. In the present case, heavy smoking may have contributed to persistent HPV infection and the development of adenocarcinoma in situ despite regular surveillance [14,15,16]. The presented case further supports the view that AIS can occur at a younger age than traditionally expected and should not be excluded from the differential diagnosis in this age group [2,16].

### 3.4. Distribution of HPV Genotypes in AIS: Beyond HPV 16 and 18

As HPV 16 and 18 account for the majority of cervical adenocarcinomas, AIS is not exclusively associated with these genotypes [4,5,17]. In addition to HPV16 and 18, other high-risk genotypes, particularly those belonging to the alpha-7 species, have been implicated in the development of glandular cervical lesions and adenocarcinoma. The alpha-7 species comprise HPV18, HPV39, HPV45, HPV59, HPV68, HPV70, HPV85 and HPV97 [15,17,18]. These genotypes exhibit oncogenic potential comparable to HPV 16 and/or 18, but are less emphasized in triage algorithms within contemporary screening programs [16,24,27]. Patients with persistent multiple high-risk HPV infections without HPV 16 or 18 positivity are often managed conservatively with prolonged follow-up, rather than immediate diagnostic intervention [24,27,29]. Although appropriate for most transient infections, this strategy may unintentionally delay the diagnosis of glandular lesions such as AIS [8,16,24]. The presented case illustrates this diagnostic blind spot, as persistent infection with multiple non-16/18 high-risk HPV types preceded definitive histopathological confirmation of AIS [17,19].

### 3.5. Cytological and Colposcopic Pitfalls in the Detection of AIS

Cytological abnormalities associated with AIS are often subtle and nonspecific [8,17]. Unlike squamous lesions, which typically produce characteristic cytological changes, glandular lesions may present with findings of ASC-US or even negative cytology [8,25]. This limited sensitivity is further exacerbated by sampling limitations, as glandular cells originating from the endocervical canal may be insufficiently represented in routine Pap tests [1,22]. Colposcopy further contributes to diagnostic uncertainty [30,34]. AIS often lacks clear colposcopic characteristics, and its endocervical location limits direct visualization of the lesion [1,20]. When abnormalities are present, they may be confined to iodine-negative or minimally acetowhite areas without clear demarcation [30,34]. A multifocal and discontinuous growth pattern increases the likelihood of false-negative biopsies, as described in our case presentation, or in cases where sampling is limited or when endocervical curettage is omitted [19,20,34].

### 3.6. Risk-Based Screening Algorithms and Diagnostic Gaps

Contemporary cervical cancer screening guidelines increasingly rely on risk stratification models in order to reduce the number of unnecessary invasive procedures [16,24,26,31,32,33]. The algorithms proposed by major professional organizations prioritize HPV 16/18 positivity and high-grade cytological abnormalities as triggers for colposcopy and biopsy [26,27,29,31]. Although effective for squamous disease, these models may underestimate the risk of glandular pathology in patients with persistent low-grade cytological abnormalities and non-16/18 high-risk HPV infection [8,16,24]. In accordance with current guidelines, in this clinical context there would be no routine indication for repeating invasive diagnostic procedures, despite persistent abnormal findings [26,27,29]. The final diagnosis of AIS following excisional treatment highlights a critical limitation of current algorithms and indicates that persistent cytological abnormalities require individualized assessment when accompanied by long-term high-risk HPV infection [8,16,24,27].

### 3.7. Fertility-Preserving Treatment and Follow-Up: Synthesis of Evidence

Management of AIS in young, nulliparous women represents a particular clinical challenge [8,19,21]. When negative margins are achieved, excisional treatment, which is most often conization, has both a diagnostic and a therapeutic purpose [8,21]. An increasing body of evidence supports fertility-preserving approaches with rigorous follow-up, with favorable oncological outcomes in carefully selected patients [19,21,37]. Follow-up strategies typically include combined HPV testing and cytology, supplemented by colposcopy and cervical sampling when indicated [8,19,27]. Persistent HPV positivity during follow-up represents a well-established predictor of disease recurrence, emphasizing the key role of virological surveillance [36,37]. Although conservative treatment carries a higher risk of local recurrence compared with definitive surgical treatment, available data indicate that there is no adverse impact on overall survival when strict follow-up protocols are consistently implemented [21,37,40].

## 4. Conclusions

In this case report, we have shown that cervical AIS can also occur in young patients, despite its low incidence in this age group. The particular significance of this case lies in the fact that the initial findings were subtle and nonspecific, including the presence of genital condylomas, minimal cytological abnormalities, absence of HPV 16/18 genotypes, and subtle colposcopic changes, which could have led to a delay in establishing the definitive diagnosis if clinical suspicion had not been present. Contemporary screening and diagnostic algorithms are primarily focused on the detection of squamous precancerous lesions (CIN2+/CIN3+), while glandular lesions, including AIS, often remain outside their optimal scope. This case demonstrates that strict reliance on standard triage criteria may be insufficient in the presence of persistent, low-grade, or nonspecific findings, particularly if they are accompanied by long-term high-risk HPV infection that does not include types 16 and 18. Furthermore, this additionally confirms that the absence of high-risk HPV 16 and 18 genotypes, normal or mildly altered cytology, and an unremarkable colposcopic finding do not exclude the presence of glandular pathology. The key message of this case report is that guidelines based on population-level risk assessment cannot fully replace individualized clinical judgment by agynecologist. In patients with persistent abnormalities, even when these do not reach the threshold for invasive diagnostics according to current recommendations, it is necessary to consider the broader clinical context, including the duration of findings, virological status, and repeated abnormalities. In such situations, a lower threshold for an excisional diagnostic approach may be justified in order to avoid missing glandular lesions with significant malignant potential. This case further emphasizes the importance of primary prevention through HPV vaccination and continuous education of young individuals, as well as the need to improve existing screening strategies to achieve better detection of glandular lesions. Future research should be directed toward the development of more precise triage and diagnostic tools that will enable earlier recognition of AIS, particularly in scenarios that deviate from typical patterns of squamous disease.

## Figures and Tables

**Table 1:** Distribution of the most common high-risk human papillomavirus (HPV) types in invasive cervical carcinoma, with comparison between squamous cell carcinoma and adenocarcinoma.

HPV Types: 16, 18, 45, 31, 33, 58, 52, 35, 59, 56, 51, 68, 39, 82, 73, 66 and 70			
Squamous Cell Carcinoma		Adenocarcinoma	
87.3%		76.4%	
HPV-16	46–63%	HPV 18	37–41%
HPV-18	10–14%	HPV-16	26–36%
HPV-45	2–8%	HPV-45	5–7%
HPV-31	2–7%		
HPV-33	3–5%		

## Data Availability

Data presented in this study are available from the corresponding author upon request.

## Abbreviations

The following abbreviations are used in this manuscript:

AIS	Adenocarcinoma in situ
HPV	Human papillomavirus
Pap	Papanicolaou
TZ	Transformation zone
TZ 1,2,3	Transformation zone 1,2,3
ECC	Endocervical curettage
AW	Acetowhite
ASC-US	Atypical squamous cells of undetermined significance
LBC	Liquid-based cytology
ASCCP	American Society for Colposcopy and Cervical Pathology
CIN II, III	Cervical intraepithelial neoplasia II, III
ACS	American Cancer Society
ASCO	American Society of Clinical Oncology
WHO	World Health Organization
CCA	Cancer Council Australia
EG	European Guidelines

## References

[1] Loureiro J., Oliva E. (2014). The spectrum of cervical glandular neoplasia and issues indifferential diagnosis. Arch. Pathol. Lab. Med..

[2] Singh D., Vignat J., Lorenzoni V., Eslahi M., Ginsburg O., Lauby-Secretan B., Arbyn M., Basu P., Bray F., Vaccarella S. (2023). Global estimates of incidence and mortality of cervical cancer in 2020: A base line analysis of the WHO Global Cervical Cancer Elimination Initiative. Lancet Glob. Health.

[3] Gadducci A., Guerrieri M.E., Cosio S. (2019). Adenocarcinoma of the uterine cervix: Pathologic features, treatment options, clinical outcome and prognostic variables. Crit. Rev. Oncol. Hematol..

[4] Clifford G.M., Smith J.S., Plummer M., Muñoz N., Franceschi S. (2003). Human papillomavirus types in invasive cervical cancer world wide: A meta-analysis. Br. J. Cancer.

[5] Schiffman M., Doorbar J., Wentzensen N., de Sanjosé S., Fakhry C., Monk B.J., Stanley M.A., Franceschi S. (2016). Carcinogenic human papillomavirus infection. Nat. Rev. Dis. Primers.

[6] Araldi R.P., Sant’Ana T.A., Módolo D.G., de Melo T.C., Spadacci-Morena D.D., de Cassia Stocco R., Cerutti J.M., de Souza E.B. (2018). The humanpapilloma virus (HPV)-related cancer biology: An overview. Biomed. Pharmacother..

[7] Nicolás I., Saco A., Barnadas E., Marimon L., Rakislova N., Fusté P., Rovirosa A., Gaba L., Buñesch L., Gil-Ibañez B. (2020). Prognostic implications of genotyping and p16 immunostaining in HPV-positive tumors of the uterine cervix. Mod. Pathol..

[8] Teoh D., Musa F., Salani R., Huh W., Jimenez E. (2020). Diagnosis and management of adenocarcinoma in situ of the uterine cervix. Obstet. Gynecol..

[9] Zeng Q., Feng K., Yu Y., Lv Y. (2024). Hsa_circ_0000021 sponges miR-3940-3p/KPNA2 expression to promote cervical cancer progression. Curr. Mol. Pharmacol..

[10] Ding X., Wan A., Qi X., Jiang K., Liu Z., Chen B. (2024). ZNF695, a potential prognostic biomarker, correlates with immune infiltrates in cervical squamous cell carcinoma and endocervical adenocarcinoma: Bioinformatic analysis and experimental verification. Curr. Gene Ther..

[11] Rangaraj K., Vasudevan M.T., Rangaraj S., Kumar R., Muthusami S., Alahmadi T.A., Chinnathambi A., Arulselvan P., Chaiyasut C., Bharathi M. (2025). Synergistic antiproliferative effects of EGCG and myricetin on cervical-cancer biomarkers in ME180 and SiHa cell lines. Curr. Gene Ther..

[12] de Paula Filho M.F.F., Lopes Chrisóstomo L.L., Cansanção I.F. (2024). HPV16 genomes: In silico analysis of E6 and E7 on coproteins in 20 South American variants. Curr. Genom..

[13] Curry S.J., Krist A.H., Owens D.K., Barry M.J., Caughey A.B., Davidson K.W., Doubeni C.A., Epling J.W., Kemper A.R., Kubik M. (2018). Screening for cervical cancer: U.S. Preventive Services Task Force recommendation statement. JAMA.

[14] Kayar İ., Goc G., Cetin F., Birge Ö. (2025). Impact of Smoking on Cervical Histopathological Changes in High-Risk HPV-Positive Women: A Matched Case–Control Study. Medicina.

[15] Bhatla N., Aoki D., Sharma D.N., Sankaranarayanan R. (2025). Cancer of the cervixuteri: 2025 update. Int. J. Gynecol. Obstet..

[16] Perkins R.B., Wentzensen N., Guido R.S., Schiffman M. (2023). Cervical cancer screening: A review. JAMA.

[17] Pirog E.C. (2017). Cervical adenocarcinoma: Diagnosis of human papillomavirus-positive and human papillomavirus-negative tumors. Arch. Pathol. Lab. Med..

[18] Chen Z., Schiffman M., Herrero R., De Salle R., Anastos K., Segondy M., Sahasrabuddhe V.V., Gravitt P.E., Hsing A.W., Burk R.D. (2013). Evolution and taxonomic classification of alphapapillomavirus 7 complete genomes: HPV18, HPV39, HPV45, HPV59, HPV68 and HPV70. PLoS ONE.

[19] Adolph L., Mann A., Liu X.Q., Roberts L., Robinson C., Popowich S., Dean E., Kean S., Fischer G., Altman A.D. (2024). Follow-up of women with cervical adenocarcinoma in situ treated by conization: A single centre clinical experience. Gynecol. Oncol..

[20] Ostör A.G., Duncan A., Quinn M., Rome R. (2000). Adenocarcinoma in situ of the uterine cervix: An experience with 100 cases. Gynecol. Oncol..

[21] Delli Carpini G., Cicoli C., Bernardi M., Di Giuseppe J., Giannella L., Ciavattini A. (2025). Clinical outcomes of cervical adenocarcinoma in situ according to conservative or demolitive treatment: A systematic review and meta-analysis. Cancers.

[22] Ito K., Kimura R., Konishi H., Ozawa N., Yaegashi N., Ohashi Y., Suzuki M., Kakizoe T. (2020). A comparison of liquid-based and conventional cytology using data for cervical cancer screening from the Japan Cancer Society. Jpn. J. Clin. Oncol..

[23] Bhatla N., Moda N. (2009). The clinical utility of HPV DNA testing in cervical cancer screening strategies. Indian J. Med. Res..

[24] Zampaoglou E., Boureka E., Gounari E., Liasidi P.N., Kalogiannidis I., Tsimtsiou Z., Haidich A.-B., Tsakiridis I., Dagklis T. (2025). Screening for Cervical Cancer: A Comprehensive Review of Guidelines. Cancers.

[25] Bansal B., Gupta P., Gupta N., Rajwanshi A., Suri V. (2016). Detecting uterine glandular lesions: Role of cervical cytology. CytoJournal.

[26] Perkins R.B., Guido R.S., Castle P.E., Chelmow D., Einstein M.H., Garcia F., Huh W.K., Kim J.J., Moscicki A.-B., Nayar R. (2020). 2019 ASCCP risk-based management consensus guidelines for abnormal cervical cancer screening tests and cancer precursors. J. Low. Genit. Tract Dis..

[27] Willows K., Selk A., Auclair M.H., Jim B., Jumah N., Nation J., Proctor L., Iazzi M., Bentley J. (2023). 2023 Canadian Colposcopy Guideline: A risk-based approach to management and surveillance of cervical dysplasia. Curr. Oncol..

[28] Elfström K.M., Arnheim-Dahlström L., von Karsa L., Dillner J. (2015). Cervical cancer screening in Europe: Quality assurance and organisation of programmes. Eur. J. Cancer.

[29] Cancer Council Australia (2022). Guidelines for the Management of Screen-Detected Abnormalities, Screening in Specific Populations and Investigation of Abnormal Vaginal Bleeding.

[30] Burness J.V., Schroeder J.M., Warren J.B. (2020). Cervical colposcopy: Indications and risk assessment. Am. Fam. Physician.

[31] Fontham E.T.H., Wolf A.M.D., Church T.R., Etzioni R., Flowers C.R., Herzig A., Guerra C.E., Oeffinger K.C., Shih Y.T., Walter L.C. (2020). Cervical cancer screening for individuals at average risk: 2020 guideline update from the American Cancer Society. CA Cancer J. Clin..

[32] Shastri S.S., Temin S., Almonte M., Basu P., Campos N.G., Gravitt P.E., Gupta V., Lombe D.C., Murillo R., Nakisige C. (2022). Secondary prevention of cervical cancer: ASCO resource-stratified guideline update. JCO Glob. Oncol..

[33] World Health Organization (2021). WHO Guideline for Screening and Treatment of Cervical Pre-Cancer Lesions for Cervical Cancer Prevention.

[34] McGee A.E., Alibegashvili T., Elfgren K., Frey B., Grigore M., Heinonen A., Jach R., Jariene K., Kesic V., Küppers V. (2023). European consensus statement on expert colposcopy. Eur. J. Obstet. Gynecol. Reprod. Biol..

[35] von Karsa L., Arbyn A., De Vuyst H., Dillner J., Dillner L., Franceschi S., Patnick J., Ronco G., Segnan N., Suonio E., Anttila A., Arbyn A., De Vuyst H.Y., Dillner J., Dillner L., Franceschi S., Patnick J., Ronco G., Segnan N., Suonio E. (2015). Executive summary. European Guidelines for Quality Assurance in Cervical Cancer Screening.

[36] Costa S., Negri G., Sideri M., Santini D., Martinelli G., Venturoli S., Pelusi C., Syrjanen S., Syrjanen K., Pelusi G. (2007). Human Papillomavirus (HPV) Test and Pap Smear as Predictors of Outcome in Conservatively Treated Adenocarcinoma In Situ of the Uterine Cervix. Gynecol. Oncol.

[37] Giannella L., Delli Carpini G., Di Giuseppe J., Grelloni C., Bogani G., Dri M., Sopracordevole F., Clemente N., Giorda G., De Vincenzo R. (2024). Long-term follow-up outcomes in women with in situ/microinvasive adenocarcinoma of the uterine cervix undergoing conservative treatment. Cancers.

[38] Matsumoto K., Yaegashi N., Iwata T., Yamamoto K., Aoki Y., Okadome M., Ushijima K., Kamiura S., Takehara K., Horie K. (2019). Reduction in HPV16/18 among young women with CIN2–3/AI Safter vaccination. Cancer Sci..

[39] Centers for Disease Control and Prevention (CDC) (2025). Trends in cervical precancers identified through population-based surveillance—Human Papillomavirus Vaccine Impact Monitoring Project, fivesites, United States, 2008–2022. Morb. Mortal. Wkly. Rep..

[40] Drolet M., Bénard É., Pérez N., Brisson M., HPV Vaccination Impact Study Group (2019). Population-level impact and herd effects following the introduction of human papillomavirus vaccination programmes: Updated systematic review and meta-analysis. Lancet.

